# Effect of motion inputs on the wear prediction of artificial hip joints

**DOI:** 10.1016/j.triboint.2012.05.029

**Published:** 2013-07

**Authors:** Feng Liu, John Fisher, Zhongmin Jin

**Affiliations:** a Institute of Medical and Biological Engineering, School of Mechanical Engineering, University of Leeds, Leeds LS2 9JT, UK; b School of Mechanical Engineering, Xi'an Jiaotong University, PR China

**Keywords:** Ultra-high molecular weight polyethylene, Artificial hip joints, Wear modelling, Motion inputs

## Abstract

Hip joint simulators have been largely used to assess the wear performance of joint implants. Due to the complexity of joint movement, the motion mechanism adopted in simulators varies. The motion condition is particularly important for ultra-high molecular weight polyethylene (UHMWPE) since polyethylene wear can be substantially increased by the bearing cross-shear motion. Computational wear modelling has been improved recently for the conventional UHMWPE used in total hip joint replacements. A new polyethylene wear law is an explicit function of the contact area of the bearing and the sliding distance, and the effect of multidirectional motion on wear has been quantified by a factor, cross-shear ratio. In this study, the full simulated walking cycle condition based on a walking measurement and two simplified motions, including the ISO standard motion and a simplified ProSim hip simulator motion, were considered as the inputs for wear modelling based on the improved wear model. Both the full simulation and simplified motions generated the comparable multidirectional motion required to reproduce the physiological wear of the bearing *in vivo*. The predicted volumetric wear of the ProSim simulator motion and the ISO motion conditions for the walking cycle were 13% and 4% lower, respectively, than that of the measured walking condition. The maximum linear wear depths were almost the same, and the areas of the wear depth distribution were 13% and 7% lower for the ProSim simulator and the ISO condition, respectively, compared with that of the measured walking cycle motion condition.

## Highlights

► Computational wear modelling was conducted for UHMWPE hip joints. ► The loading/motion of the measured walking, the ISO and the ProSim simulator was analysed. ► The cross-shear ratios, slide tracks, and contact pressures were compared. ► The comparable wear was predicted.

## Notation

*d*: thickness of polyethylene cup wall
$\bar{p}$: average contact pressure over loading history
*t*: time of loading history in minutes
*A*: nominal contact area
*B*: creep constant
*C*: wear coefficient
*CSR*: cross-shear ratio
*L*: sliding distance
*V*: wear volume
*W*_*pi*_: discretised frictional work in the PMO direction
*W*_*ti*_: discretised frictional work perpendicular to the PMO direction
*δ*: linear wear depth
*δ*_*cr*_: creep deformation at a point

## 1 Introduction

Artificial joint replacements are effective in providing normal function to many patients suffering from severe joint diseases [1]. The joint replacement treatment has been continuously evolved, from hips and knees to other major synovial joints [2], [3], [4], [5]. However, the joint bearings are subject to wear. Wear debris generated mainly from the joint bearing surface accumulates in local tissues, causes adverse tissue reaction, and ultimately leads to implant fixation failure [6]. Wear-induced failure remains a major limiting factor affecting the long-term performance of the joint replacements, particularly for younger and more active patients. Recognition of the wear issue has led to extensive wear studies to predict wear performance, understand wear mechanism and evaluate design factors [7], [8], [9], [10].

Wear studies of artificial hip joint bearings have been largely carried out experimentally. Wear tests based on a simple bearing configuration using pin-on-plate testers are useful for identifying wear properties. For example, conventional UHMWPE has been substantially tested to determine variations in wear rates with changes in individual tribological system parameters such as contact pressure, cross-shear, or counterface roughness [11], [12], [13], [14]. The tests have been extended to develop wear laws for polyethylene joint bearings [15]. For the full-scale wear simulation of an actual joint bearing, joint simulators have been developed to replicate the motion, loading and environment *in vivo* [16]. However, a precise reproduction of the complex operating conditions is generally difficult [17]. The hip simulation ISO standard for wear assessment defines a standard walking cycle for a standard patient [18]. In many hip simulator designs, kinematics and loading conditions have been further simplified such as by reducing the full three axes rotation to flexion/extension and internal/external rotation [16]. With increasing demands for the implant wear testing under various functional requirements, the simulators that can provide a balanced complexity and accuracy are necessary, and such a design requires a better understanding of the effects of various operating conditions on wear. As an alternative, computational wear modelling is a suitable means to provide a rapid and vigorous comparison between multiple variables in a parametric study [19], [20], [21], [22], [23].

Computational wear modelling has improved for polyethylene artificial joints. Brown et al. [24] have pioneered the computational wear simulation of joint implants based on Archard's law and finite element contact models. The approach has been widely applied [25], [26], [27], [28], [29], and continually enhanced particularly in an attempt to develop a generic wear model for UHMWPE based on fundamental wear properties [15], [30]. Recent advances include the quantification of multidirectional motion effect on polyethylene wear [13], and the contact area dependent wear law [15], [31] in which the wear volume is proportional to the nominal contact area and sliding distance. The wear law assumes that the wear coefficient is constant over contact pressures but a function of the cross-shear resulting from the multidirectional motion. The use of the new model has therefore provided an independent wear prediction and improved the prediction agreement with that of experimental tests for both polyethylene hip and knee joints [15], [31]. The wear modelling has been further improved to include polyethylene creep in a parametric study on the joint diameters and bearing clearances of polyethylene hip joints [32].

Motion inputs are a major factor in the simulator wear studies [16], [17]. Saikko and Calonius [33] have shown considerable differences in slide tracks on acetabular cup bearings resulting from varied motions, which implies a possible reason for causing a large difference in polyethylene wear. The present study focussed on the computational wear prediction of polyethylene cup bearings based on the contact area dependent wear law, and considered three types of motion inputs, the measured walking cycle [34], ISO 14242 recommendation [35], and simplified ProSim simulator testing [36] to justify the simplification of the motion inputs to provide guidance to the simulator design and testing.

## 2 Materials and methods

**Fig. 1:**
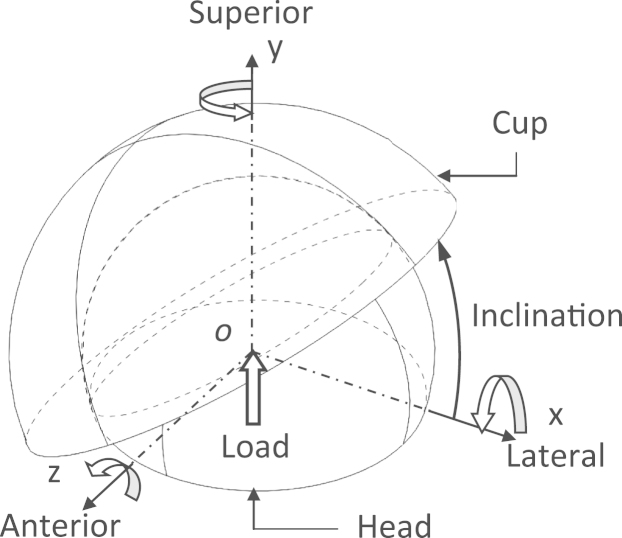
Schematic of three-dimensional total hip joint with load and motion of flexion/extension about the *x*-axis, abduction/adduction about the *y*-axis and internal/external rotation about the *z*-axis.

Total hip joint replacements consisting of an acetabular cup and a femoral head were modelled as a simple ball-in-socket configuration, as schematically shown in Fig. 1, with respect to a fixed rectangular Cartesian coordinate system (*OXYZ*) [26], [32]. The bearing materials were chosen as the conventional UHMWPE GUR 1050 (non-cross-linked) for the cup and cobalt chrome alloy (CoCrMo) for the head. In the present study, the cup was inclined at 35° under a vertically applied resultant load, which replicates a general cup inclination of 45° in the pelvis with the load vector 10° medially [37]. A nominal 28 mm diameter hip joint with a radial clearance of 0.04 mm and the cup wall thicknesses of 8.0 mm and 4.0 mm, respectively, for the polyethylene bearing and its metallic backing shell was analysed [15]. The bearing materials used in this study are summarised in Table 1.

**Table 1:** Details of the range of motion and moving components for the three cases considered in this study, the measured walking, ISO 14242 and ProSim simulator conditions.

Simulation conditions	Flexion/extension		Abduction/adduction		Internal/external rotation	
	Range (deg.)	Moving bearing	Range (deg.)	Moving bearing	Range (deg.)	Moving bearing
Case 1: Walking	30/−15	Head	5/−4	Head	6/−8	Head
Case 2: ISO	25/−18	Cup	4/−7	Cup	2/−10	Head
Case 3: ProSim	30/−15	Head			10/−10	Cup

**Fig. 2:**
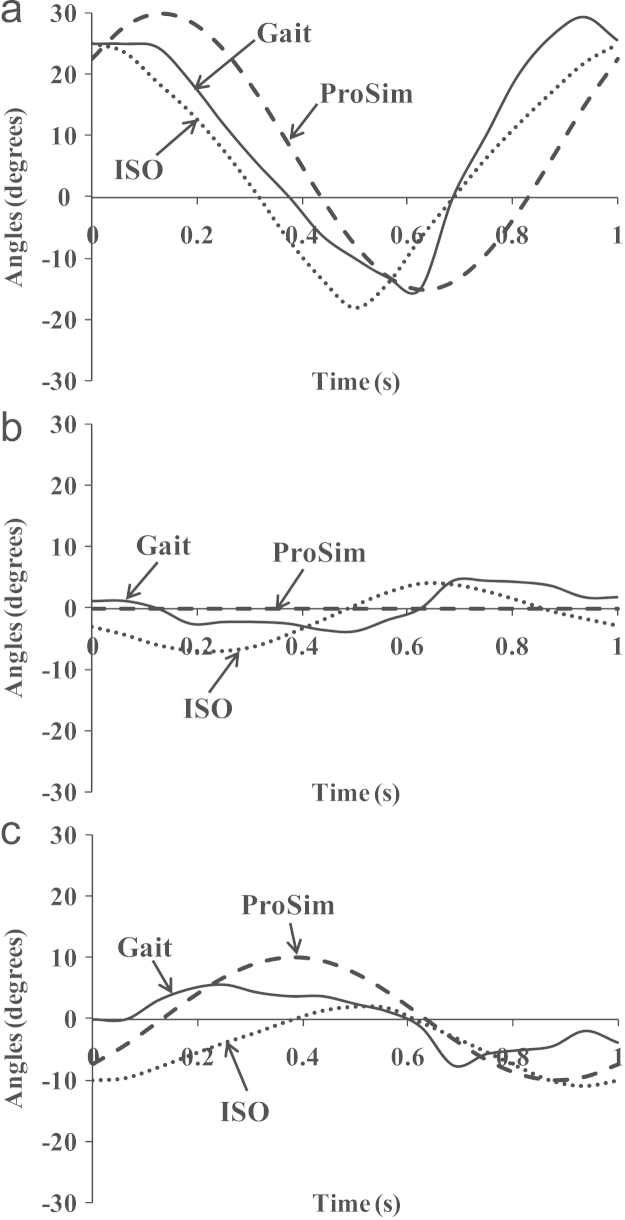
Motion profiles of (a) flexion/extension, (b) abduction/adduction and (c) internal/external rotation for the measured walking cycle, ISO standard and ProSim simulator (two-axis) conditions.

**Fig. 3:**
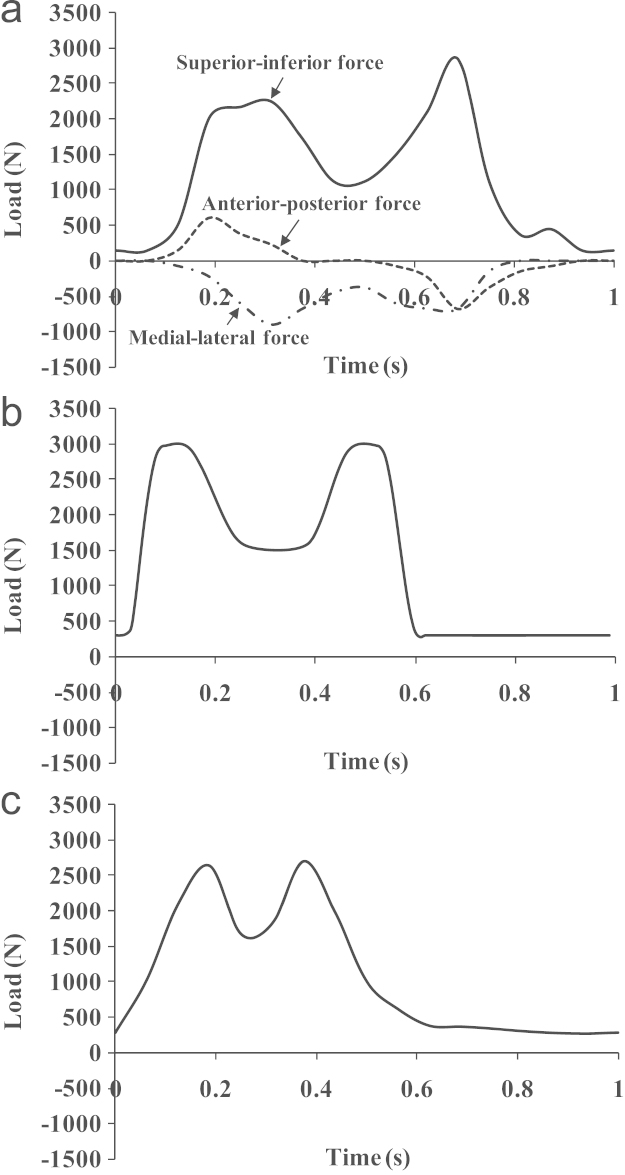
Loading profiles of (a) the measured walking cycle, (b) ISO standard and (c) ProSim simulator conditions.

Three sets of motion and loading conditions were considered to represent a physiological walking cycle of hip joints; the full simulated condition based on a gait measurement [34], [19], and the simplified conditions including the ISO [35] and ProSim simulator testing [36], as shown in Fig. 2, Fig. 3 for motion and loading profiles over a cycle, respectively. All the rotation components given above were considered as Euler angles; the rotation transformation was performed following the sequence of flexion/extension (FE), abduction/adduction (AA) and internal/external rotation (IER), and the rotation movements were performed on different bearing components for different cases [33]. For the measured walking (Case 1), all three rotations of FE, AA and IER were carried by the head with the cup being stationary; for the ISO condition (Case 2), the FE and AA rotations were conducted on the cup, and the IER rotation by the head; In Case 3, the simplified ProSim simulator testing, the FE and IER rotations were achieved on the cup and head, respectively. The loading was generally considered to be similar with the magnitudes of the averaged loads being, respectively, 1242, 1293 and 1057 N for the above Cases 1, 2 and 3, and the resultant load directions being similarly varied relative to the cup bearing surface [19], [35], [36]. The details of the range of motions and the associated moving bearings considered are summarised in Table 2 for the measured walking cycle, ISO and ProSim simulator [35], [36].

**Table 2:** Details of the predicted maximum linear depth, wear distribution area, maximum cross-shear ratio, and sliding distance averaged over whole bearing surface for a motion cycle, of a 28 mm diameter bearing surface after 5 million cycles for the three conditions considered, the measured walking, ISO and ProSim simulator conditions.

	Max. linear wear depth (mm)	Wear distribution area (mm^2^)	Max. cross-shear ratio	Average sliding distance (mm)
Case 1: Walking	0.1	971	0.35	20
Case 2: ISO	0.09	902	0.3	18
Case 3: ProSim	0.09	842	0.35	21

The details of computational wear modelling for conventional UHMWPE bearings have been previously published, which include the development of the contact area dependent wear law [15], the quantification of the cross-shear effect [15], [31], and the full numerical calculation of wear and creep [32]. The methods were directly applied in this study, and for clarity, the major equations are briefly described in this paper. The volumetric wear was expressed as

(1) V=CAL

where *A* is the contact area, *L* is the sliding distance and *C* is the wear coefficient [15]. The linear wear depth (*δ*) was derived from Eq. (1) by dividing the contact area as

(2) δ=CL

The wear coefficient (*C*) was assumed to be constant over the contact pressure range as experienced in the joint replacements, but dependent on the cross-shear resulting from the multidirectional sliding motion of the bearings. A general theory on the cross-shear effect of polyethylene wear is based on the polyethylene molecular reorientation and strain hardening [38], [39]. The polyethylene molecules on the bearing surface align with the principal direction of sliding, defined as principal molecular orientation (PMO), and lead to strain hardening and enhanced wear resistance; the sliding against the principal direction weakens the molecular reorientation and causes more wear. The cross-shear was quantified using a cross-shear ratio, *CSR*, given by

(3) $$CSR=\sum W_{ti}/\sum \left(W_{ti}+W_{pi}\right)$$

where *W*_*ti*_ and *W*_*pi*_ are the discretised frictional work resolved in two directions, perpendicular to and along with the PMO of the bearing, respectively. The frictional work was calculated as the product of frictional force and sliding distance, and contact pressure determined from the bearing contact model was used to calculate the frictional force. The PMO was considered to be the direction along which the maximum amount of frictional work is released, and was numerically determined by iteratively comparing all the possible PMO directions [15], [32].

The ram-extruded non-cross-linked UHMWPE (0 Mrad, GUR 1050) pin and the cobalt–chromium plate with an average surface roughness *R*_a_ of approximately 0.01 μm was used to determine the wear coefficient with pin-on-plate wear testing under conditions of the multidirectional motion, varied contact pressures and lubricated environment replicating those of full joint simulation [19]. The cross-shear was achieved by varying the rotation of polyethylene pins and the translation of metal plates in tests. The test was carried out at normal laboratory room temperature (approximately 20°) with 25% solution of new-born calf serum as the lubricant. The details of the pin-on-plate wear test can be found elsewhere [19]. The curve-fitted general wear coefficient (*C*) as a function of cross-shear ratio for the conventional non-cross-linked UHMWPE (GUR 1050) for contact pressure range considered (1–10 MPa) was given by

(4) $$C={\left(8.5\times 10^{-5}+9.3\times CSR\right)}^{0.15}\times 10^{-60}$$

A finite element contact model was constructed with linear hexahedral elements for the cup and an analytical rigid body for the head and solved to obtain contact pressure using ABAQUS^™^ (version 6.8-1, SIMULIA, Rhode Island) [15]. Approximately, 2000 elements were used for the polyethylene cup with a typical element length of 0.5 mm to ensure converged contact stress. The polyethylene with Young's modulus, 500 MPa, and Poisson's ratio, 0.4, was modelled as an elastic–plastic material, with initial Von Mises yield stress of 10.8 MPa as tested by Barbour et al. [12] using isotropic GUR412 UHMWPE (molecular weight 4×10^6^ g/mol). This data was assumed to be similar to that of the GUR 1050 in terms of elastic–plastic properties considered in the present study.

The motion/loading cycle of 1 s was equally discretised into 16 time points. At each time point, the contact model was solved. For each nodal point on the cup bearing surface, the contact pressures obtained over the loading cycle were used to determine the wear coefficient, and subsequently the linear wear depths at each time point and the accumulated linear wear over the cycle as

(5) $$\delta =\sum_{i=1}^{n}C(CSR)\cdot L_{i}$$

where *n* is the number of discretised time points in a loading cycle, the cross-shear ratio (*CSR*), and *L*_*i*_ is the sliding distance in the *i*th interval. The wear depth calculated for a single loading/motion cycle was then scaled by a geometry update factor that is the number of loading/motion cycles in the interval after which the bearing geometry was modified. For the initial 64,000 cycles (the first 2000 min), 10 intervals evenly selected using a logarithmic scale were considered in order to calculate the high creep strain rate [32], and the geometry update factor was then fixed at 250,000 throughout the rest of simulation [24], [26]. The linear creep deformation at a geometric point (*δ*_*cr*_) was calculated as

(6) $$\delta_{cr}=B\cdot \bar{p}\cdot \log(t)\cdot d$$

where *B* is a creep constant, 7.97/[log(min)) MPa], based on the extruded, unirradiated GUR 4150HP UHMWPE rod stock under constant pressures of 2.0–8.0 MPa in a 37 °C bovine serum reservoir for the polyethylene used [40], $\bar{p}$ is the average contact pressure over loading history, *t* is the time length of the loading duration in minutes, and *d* is the thickness of the polyethylene bearing. The total linear penetration at a geometric point was considered to be the superposition of the linear wear and creep. The bearing geometry was modified by correcting the nodal coordinates of the contact model after each geometry update interval, and the finite element contact model was then recalculated based on the new geometry. The total simulation was carried out for 5 million cycles.

## 3 Results

**Fig. 4:**
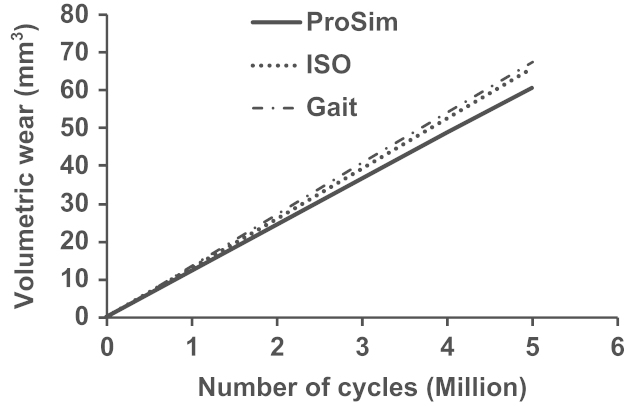
Comparison of the accumulated volumetric wear predicted for the measured walking, ISO standard, and ProSim simulator conditions over the number of simulated cycles for a 28 mm diameter polyethylene cup bearing with a radial clearance of 0.04 mm. (The results for the ProSim simulator condition is based on previous calculation [32].)

The accumulated volumetric wear of the polyethylene cup bearings predicted with the three different motion inputs is compared in Fig. 4, as a function of the number of simulated cycles, with the wear rates being 14.0, 13.4 and 12.2 mm^3^ per million cycles, respectively, for the measured walking, ISO and ProSim simulator conditions. The predicted volumetric wear rates of the ISO and ProSim simulator conditions were 4% and 13% lower, respectively, compared with that of the measured walking condition.

**Fig. 5:**
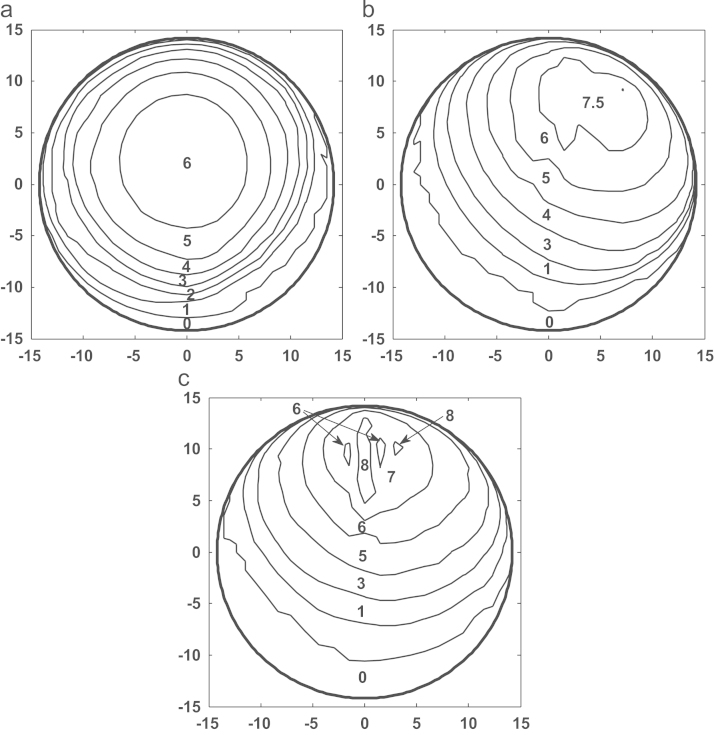
Comparison of the contact pressure distributions predicted for (a) the measured walking cycle, (b) ISO standard, and (c) ProSim simulator conditions for a 28 mm diameter polyethylene cup bearing after 5 million cycles, with the averaged contact pressure (MPa) values being illustrated. (The results for the ProSim simulator condition is based on previous calculation [32].)

**Fig. 6:**
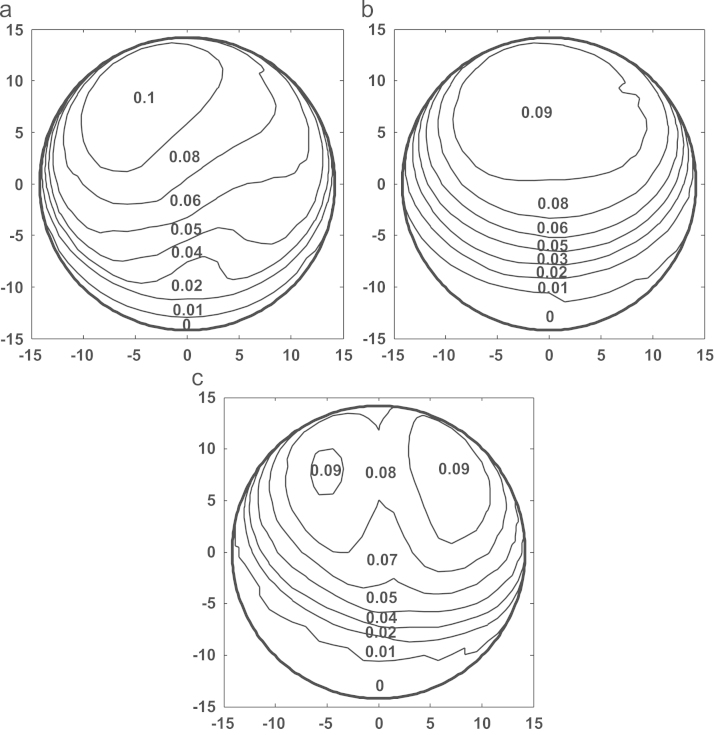
Comparison of the wear depth distributions predicted for (a) the measured walking cycle, (b) ISO standard, and (c) ProSim simulator conditions for a 28 mm diameter polyethylene cup bearing after 5 million cycles, with the averaged wear depth (mm) values being illustrated (The results for the ProSim simulator condition is based on previous calculation [32].)

**Fig. 7:**
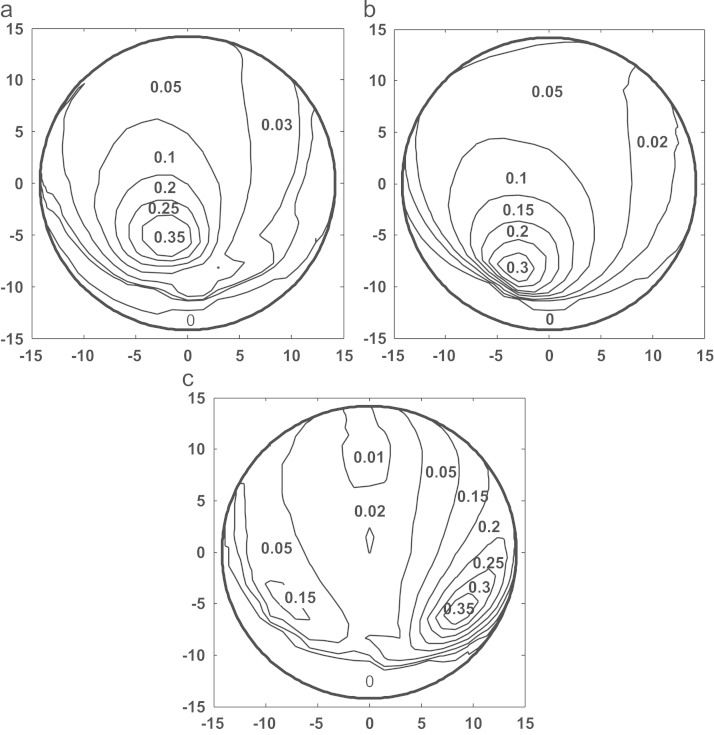
Comparison of the cross-shear ratio distributions predicted for (a) the measured walking cycle, (b) ISO standard, and (c) ProSim simulator conditions for a 28 mm cup bearing after 5 million cycles, with the averaged cross-shear ratio values being illustrated.

Fig. 5a–c shows the comparison of contact pressure distributions predicted on the bearing surface corresponding to the first peak loads of the three simulation conditions after 5 million cycles. The contact pressures predicted were generally comparable in terms of distribution areas and values, with slightly varied peak values of 6.0, 7.5 and 8.0 MPa for the measured walking, ISO and ProSim simulator conditions, respectively, and slightly different locations on the bearings (Fig. 5a–c). The comparisons of wear depths and cross-shear ratios after 5 million cycle simulation are shown in Fig. 6, Fig. 7 respectively. The worn area for the measured walking condition was found to be larger by 7% and 13% (Table 2), respectively, compared with those of the ISO and ProSim simulator conditions, but the maximum linear wear depths were approximately 0.1 mm for all cases (Fig. 6). A good agreement in linear wear depths occurred between the three axes rotation motions of the measured walking and ISO conditions (Fig. 6a and b), while the use of the two axes of rotation led to the greater wear depths at two separate locations on the bearing (Fig. 6c). The above trends in linear wear depths were mainly attributed to the cross-shear ratios, and good agreement was also found between the measured walking and ISO conditions (Fig. 7a and b), and the two axes of rotation of the ProSim simulator led to the large cross-shear ratios at separated locations (Fig. 7c). However, the cross-shear ratios were generally comparable in terms of distribution sizes and value ranges between 0 and 0.35 for all conditions despite of the double peaks under the ProSim simulator condition. Moreover, for the non-cross-linked conventional UHMWPE, wear coefficients increased with the cross-shear ratio raised to the power of 0.15, as given in Eq. (4), which showed a sharp increase (3.2–7.7×10^−10^) over low cross-shear ratios (*CSR*=0–0.01) and were steady (1.0–1.6×10^−9^) over large ratios (*CSR*=0.02–0.5). The major cross-shear ratio range of 0.02–0.4 was predicted on the polyethylene cup bearings (Fig. 7a–c) and the resulting wear coefficients were generally constant (1.0–1.6×10^−9^) over the bearing surfaces.

**Fig. 8:**
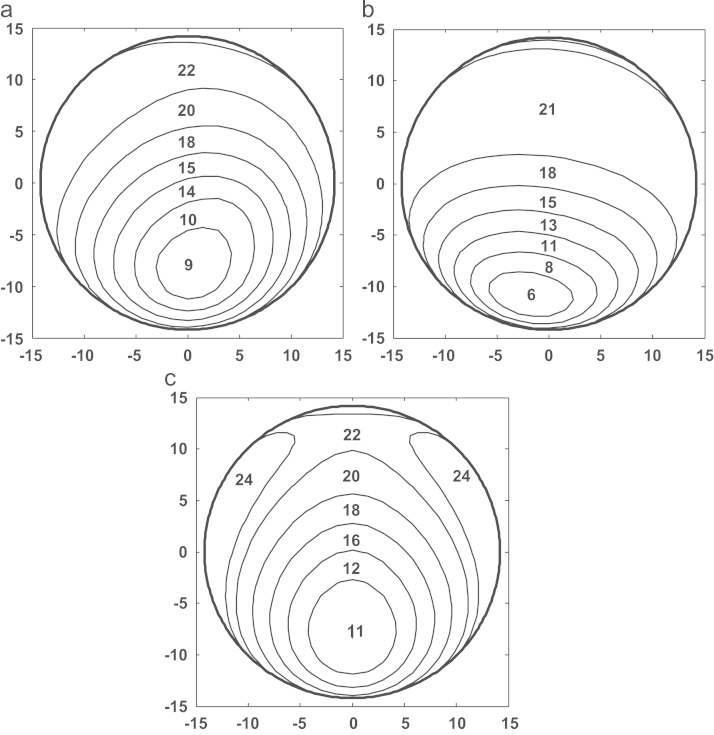
Comparison of the sliding distance distributions predicted for (a) the measured walking cycle, (b) ISO standard, and (c) ProSim simulator conditions for a 28 mm cup bearing over a motion cycle, with the averaged values being illustrated (mm).

Fig. 8a–c shows the comparison of sliding distance distributions computed for the cup bearing surfaces. The sliding distances around the main loading areas on cup bearing surfaces were found to be in a comparable range, 9–22, 8–21, and 11–22 mm, respectively, for the three cases. The maximum linear wear depths, the areas of wear distribution, the maximum cross-shear ratios, and the average sliding distances are summarised in Table 3.

**Table 3:** Materials for the joint bearings considered in experimental wear tests including joint simulators and pin-on-plate tester, and material properties used for computational modelling.

Components	Material	Young's modulus (MPa)	Poisson's ratio
Acetabular cup/polyethylene pin	Conventional UHMWPE	500	0.4
Femoral head/metal plate	Cobalt-chrome alloy	210,000	0.3
Cup backing shell	Titanium	210,000	0.3

**Fig. 9:**
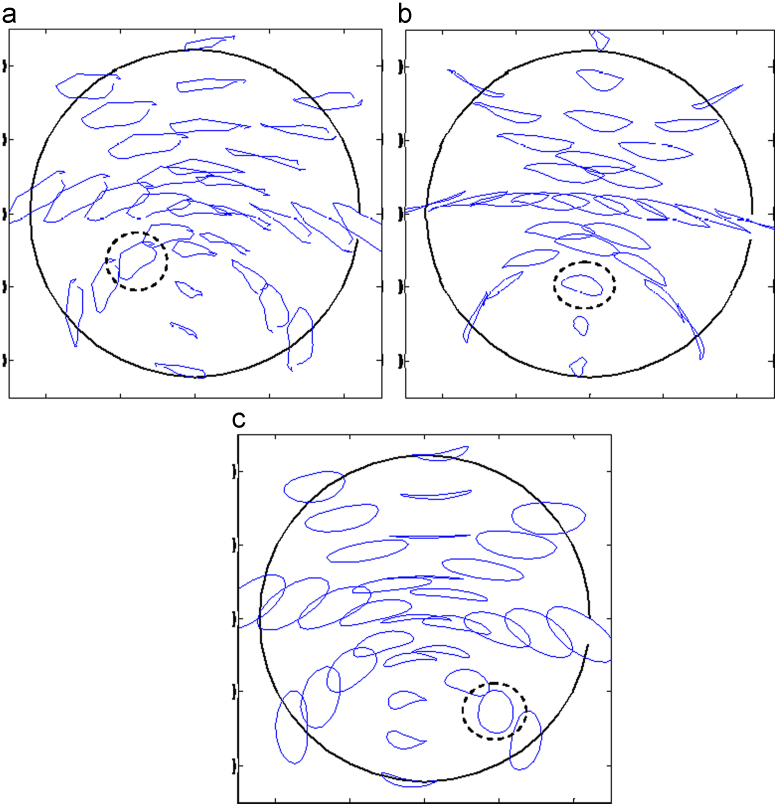
Comparison of the slide tracks of the points on the cup bearing surface relative the head bearing surface calculated for (a) the measured walking, (b) ISO standard, and (c) ProSim simulator conditions, for a 28 mm diameter cup bearing (the hemisphere bearing surface is flattened).

The slide tracks of some representative points on the cup bearing surface were computed with respect to the head bearing surface and shown in Fig. 9a–c, for the three conditions. In Fig. 9, the hemispherical bearing surface was flattened in order to show the slide track accurately without much distortion in a projection plot [33]. Therefore, the radial distance of the flattened surface equals to the distance measured from the pole along the spherical surface to the equator of the hemisphere; for example, the radius of the flattened surface is 22 mm in the present plot (Fig. 9). The plotted slide tracks are similar to those of Saikko and Calonius [33]. The tracks were less smooth for the measured walking condition due to the less smooth motion inputs from the measurement (Fig. 9a).

## 4 Discussion

Previous experimental studies carried out to investigate the effect of motion inputs on wear using ProSim simulators showed comparable volumetric wear. Barbour et al. [12] reported a typical wear rate of 38 mm^3^ per million cycles for the three-axis rotation motion and a slightly lower rate of 32 mm^3^ per million cycles by Bigsby et al. [41] for the two-axis rotation motion. In this study, the corresponding wear rates were 14 and 12 mm^3^ per million cycles, respectively, for the three-axis and two-axis rotation conditions (Fig. 4). In addition to the wear rate, the trend that the wear is comparable between the full and simplified rotation conditions was predicted by the present model. As pointed out in previous studies on model development [15], the lower wear prediction was mainly due to the underestimation of the wear coefficients. The pin-on-plate wear tests were conducted under constant load other than dynamic as in joint simulators. However, the multidirectional motion of the bearings, the resulting cross-shear and its effect on wear were fully modelled, quantified and incorporated based on pin-on-plate wear tests to study the effects of motion inputs. The contact pressure distributions predicted with respect to the peak loads at 5 million cycles were generally comparable (Fig. 5) under all the three simulation conditions. The linear wear depth distributions generally followed the contact pressure distributions (Fig. 6). The greater wear depths, for example, 0.08–0.1 mm, occurred at the major loading areas on the bearing surfaces (Fig. 6a–c) showing the dominant effect of loading in determining the contact area and consequently wear. The distributed linear wear for the three simulation conditions was generally comparable in terms of the distribution area and the range of wear depth values, which led to the comparable volumetric wear (Fig. 4).

Based on the multidirectional theory and the assumption that both the molecular reorientation and polyethylene wear are a result of dissipated frictional energy, the cross-shear was quantified as the proportion of frictional work perpendicular to the PMO to the total frictional work [13], [15]. For UHMWPE cup bearings, in addition to loading, variations in motion inputs, which can affect cross-shear ratio, wear coefficients, and sliding distance, would be the major factor affecting wear as discussed below.

The cross-shear ratios for all the three simulation conditions markedly varied between 0.01 and 0.35 over the bearing surfaces (Fig. 7), indicating that complicated cross-shear sliding occurred for both the three axes rotation and the simplified two axes rotation. The cross-shear ratios resulting from the three axes rotation of the measured walking and ISO were in good agreement (Fig. 7a and b), while the two axes rotation of the ProSim simulator led to a major variation in cross-shear ratios with the double-peak distribution (Fig. 7c) compared to that with single peak for the three axes rotation conditions. The double-peaked cross-shear ratio led to the double peak distribution of wear depths as demonstrated by the maximum wear depths being predicted at two separate locations on the bearing surface (Fig. 6c). However, the cross-shear ratios for all the three simulation conditions were generally in the range of 0.02–0.2 for the points at the main contact areas on the bearing, and the larger cross-shear ratios (*CSR*>0.2) were generally distributed close to the peripheries of the main contact areas (Fig. 7a–c). Therefore, the effect of the larger cross-shear ratios (*CSR*>0.2) on wear was considerably reduced due to the lack of contact in generating wear compared to those points within the main contact loading areas.

The sliding distances of the points on cup bearing surfaces over a single motion cycle varied in a wide range of 6–24 mm with the averages being approximately 20, 18, 21 mm, respectively, for the measured walking, ISO and ProSim conditions (Fig. 8a–c). However, the larger sliding distances were found to occur mainly at the main contact areas with comparable values ranging from 15 to 22 mm for all the motion inputs, and consequently the larger linear wear depths in these areas in addition to the effects of loading and cross-shear. The computed slide tracks of cup bearing surfaces (Fig. 9a–c) were found to be closely linked with the cross-shear ratios (Fig. 7a–c). In contrast to the linear tracks leading to lower cross-shear ratios, the open slide tracks resulted in larger cross-shear ratios. Therefore, particularly for the ProSim condition, the distributed slide tracks on the bearing surface gradually changing shapes from elliptical to linear in the central region of the distribution (Fig. 9c) led to the decreased cross-shear ratios for those points near the centre and consequently the double peak distribution (Fig. 7c). Additionally, for the maximum cross-shear ratios under all the three conditions, 0.3 or 0.35 (Fig. 7) the corresponding slide tracks are comparable in the elliptical shapes as highlighted in the example in Fig. 9a–c.

In hip joint wear simulation of a normal walking condition, motion inputs can be simplified to lead to generally comparable wear as illustrated by the three cases considered in this study. The ISO condition closely replicated the three rotations of the measured walking, despite slight differences in the phases of AA and IER rotations; the simplified ProSim simulator motion was considered to be generally comparable with three rotation motion with AA rotation being removed and IER rotation being slightly increased in amplitudes approximately by 30% (Fig. 2b and c). The computational modelling clearly demonstrated the complex cross-shear effect of bearing motion on major parameters in affecting wear such as slide tracks, sliding distances, cross-shear ratios, and wear coefficients. However, the resulting variations in major parameters were generally in a comparable range. The overall volumetric wear was therefore not largely affected by motion inputs. Normal walking is obviously only one of the joint activities. Even under similar activities, both motion and loading conditions can be significantly different for different patients, which can be expected to have more significant impact on wear and should be analysed further. Again, the wear coefficient determination based on the pin-on-plate wear testing to represent the full testing conditions including loading and motion was considered with some limitations. A recent study has shown the possible dynamic loading effect on UHMWPE wear [42]. It has also been recognised that UHMWPE-on-metal hip implants are working under boundary and mixed lubrication condition [43]. The effect of lubrication on wear was only generally incorporated in the wear coefficient by using the same lubricant in the pin-on-plate testing as in the simulator. The Young's modulus of 500 MPa used for UHMWPE in this study was lower than that of UHMWPE after *in vivo* oxidation as considered in other studies [24], [25]. The use of lower Young's modulus can generally lead to larger bearing deformation prediction, but its effect on wear was considered to be small in the present modelling. Additionally, the strain softening effect on wear has been improved recently in which Lee et al. [44] pointed out that each sliding component may have an individual softening factor to provide better quantification of the cross-shear effect. All these limitations need further considerations.

## 5 Conclusions

The effect of motion inputs on UHMWPE wear was quantitatively analysed based on the major parameters in determining wear including cross-shear ratios, sliding distances, slide tracks and wear coefficients. Both the full simulated motion condition using the walking measurement, and the simplified motion simulation, including ISO standard and ProSim simulator conditions generated markedly varied but comparable cross-shear effect on wear over the cup bearing surfaces. The major range of cross-shear ratios (0.02–0.2) was found in the main contact areas on the bearings, and the larger cross-shear ratios (*CSR*>0.2) were generally at the peripheries of the main loading areas. The predicted volumetric wear and linear wear depths were generally comparable under all the three sets of simulation conditions. The present study supports the use of the simplified motions of the ISO and the ProSim simulator to simulate the physiological walking conditions in wear prediction.

## References

[1] Fisher J., Jin Z., Tipper J.L., Stone M.H., Ingham E. (2006). Presidential guest lecture: tribology of alternative bearings. Clinical Orthopaedics and Related Research.

[2] Fisher J., Jennings L.M., Galvin A.L., Jin Z., Stone M., Ingham E. (2010). Knee society presidential guest lecture: polyethylene wear in total knees. Clinical Orthopaedics and Related Research.

[3] Hall RM, Brown TD, Fisher J, Ingham E, Mendoza SA, Mayer HM. Introduction to lumbar total disc replacement: factors that affect tribological performance. Proceedings of the Institution of Mechanical Engineers: Part J, Journal of Engineering Tribology 2006;220:775–786.

[4] Gill L.H. (2004). Challenges in total ankle arthroplasty. Foot & Ankle International.

[5] Nam D., Kepler C.K., Nho S.J., Craig E.V., Warren R.F., Wright T.M. (2010). Observations on retrieved humeral polyethylene components from reverse total shoulder arthroplasty. Journal of Shoulder and Elbow Surgery/American Shoulder and Elbow Surgeons.

[6] Ingham E., Fisher J. (2000). Biological *reactions* to wear debris in total joint replacement. Proceedings of the Institution of Mechanical Engineers: Part H, Journal of Engineering in Medicine.

[7] Clarke I.C. (1981). Wear of artificial joint materials IV Hip joint simulator studies. Engineering in Medicine.

[8] MeKellop H., Clarke I., Markolf K., Amstutz H. (1981). Friction and wear properties of polymer, metal and ceramic prosthetic joint materials evaluated on a multi-channel screening devices. Journal of Biomedical Materials Research.

[9] Hammerberg E.M., Wan Z., Dastane M., Dorr L.D. (2010). Wear and range of motion of different femoral head sizes. Journal of Arthroplasty.

[10] Livermore J., Ilstrup D., Morrey B. (1990). Effect of femoral head size on wear of the polyethylene acetabular component. Journal of Bone & Joint Surgery.

[11] Saikko V. (2006). Effect of contact pressure on wear and friction of ultra-high molecular polyethylene in multidirectional sliding. Proceedings of the Institution of Mechanical Engineers: Part H, Journal of Engineering in Medicine.

[12] Barbour P.S.M., Barton D.C., Fisher J. (1995). The influence of contact stress on the wear of UHMWPE for total replacement hip prostheses. Wear.

[13] Kang L., Galvin A.L., Brown T.D., Jin Z., Fisher J. (2008). Quantification of the effect of cross-shear on the wear of conventional and highly cross-linked UHMWPE. Journal of Biomechanics.

[14] Cooper J.R., Dowson D., Fisher J. (1993). Macroscopic and microscopic wear mechanisms in ultra-high molecular weight polyethylene. Wear.

[15] Liu F., Galvin A.L., Jin Z., Fisher J. (2011). A new formulation for the prediction of polyethylene wear in artificial hip joints. Proceedings of the Institution of Mechanical Engineers: Part H, Journal of Engineering in Medicine.

[16] Barbour P.S.M., Stone M.H., Fisher J. (1999). A hip joint simulator study using simplified loading and motion cycles generating physiological wear paths and rates. Proceedings of the Institution of Mechanical Engineers: Part H, Journal of Engineering in Medicine.

[17] Goldsmith A.A., Dowson D. (1999). Development of a ten-station, multi-axis hip joint simulator. Proceedings of the Institution of Mechanical Engineers: Part H, Journal of Engineering in Medicine.

[18] ISO Standard 14242-1:2002. Implants for surgery-wear of total hip joint prostheses. Part I: loading and displacement parameters for wear testing machines and corresponding environmental conditions for test (International Standardization Organization, Switzerland).

[19] Kang L., Galvin A.L., Jin Z., Fisher J. (2006). A simple fully integrated contact-coupled wear prediction for ultra-high molecular weight polyethylene hip implats. Proceedings of the Institution of Mechanical Engineers: Part H, Journal of Engineering in Medicine.

[20] Bouchard S.M., Stewart K.J., Pedersen D.R., Callaghan J.J., Brown T.D. (2006). Design factors influencing performance of constrained acetabular liners: finite element characterization. Journal of Biomechanics.

[21] Jourdan F., Samida A. (2009). An implicit numerical method for wear modeling applied to a hip joint prosthesis problem. Computer Methods in Applied Mechanics and Engineering.

[22] Ong K.L., Rundell S., Liepins I., Laurent R., Markel D., Kurtz S.M. (2009). Biomechanical modeling of acetabular component polyethylene stresses, fracture risk, and wear rate following press-fit implantation. Journal of Orthopaedic Research : Official Publication of the Orthopaedic Research Society.

[23] Strickland M.A., Dressler M.R., Taylor M. (2012). Predicting implant UHMWPE wear in-silico: a robust, adaptable computational–numerical framework for future theoretical models. Wear.

[24] Maxian T.A., Brown T.D., Pedersen D.R., Callaghan J.J. (1996). The Frank Stinchfield Award. 3-Dimensional sliding/contact computational simulation of total hip wear. Clinical Orthopaedics and Related Research.

[25] Bevill S.L., Bevill G.R., Penmetsa J.R., Petrella A.J., Rullkoetter P.J. (2005). Finite element simulation of early creep and wear in total hip arthroplasty. Journal of Biomechanics.

[26] Liu F., Leslie I., Williams S., Fisher J., Jin Z. (2008). Development of computational wear simulation of metal-on-metal hip resurfacing replacements. Journal of Biomechanics.

[27] Matsoukas G., Willing R., Kim I.Y. (2009). Total hip wear assessment: a comparison between computational and *in vitro* wear assessment techniques using ISO 14242 loading and kinematics. Journal of Biomechanical Engineering.

[28] Goreham-Voss C.M., Hyde P.J., Hall R.M., Fisher J., Brown T.D. (2010). Cross-shear implementation in sliding-distance-coupled finite element analysis of wear in metal-on-polyethylene total joint arthroplasty: intervertebral total disc replacement as an illustrative application. Journal of Biomechanics.

[29] Zhao D., Sakoda H., Sawyer W.G., Banks S.A., Fregly B.J. (2008). Predicting knee replacement damage in a simulator machine using a computational model with a consistent wear factor. Journal of Biomechanical Engineering.

[30] Kang L., Galvin A.L., Fisher J., Jin Z. (2009). Enhanced computational prediction of polyethylene wear in hip joints by incorporating cross-shear and contact pressure in additional to load and sliding distance: effect of head diameter. Journal of Biomechanics.

[31] Abdelgaied A., Liu F., Brockett C., Jennings L.M., Fisher J., Jin Z. (2011). Computational wear prediction of artificial knee joints based on a new wear law and formulation. JBiomechanics.

[32] Liu F., Fisher J., Jin Z. (2012). Computational modelling of polyethylene wear and creep in total hip joint replacements: effect of the bearing clearance and diameter. Proceedings of the Institution of Mechanical Engineers: Part J, Journal of Engineering Tribology.

[33] Saikko V., Calonius O. (2002). Slide track analysis of the relative motion between femoral head and acetabular cup in walking and in hip simulators. Journal of Biomechanics.

[34] Johnston R.C., Smidt G.L. (1969). Measurement of hip-joint motion during walking-evaluation of an electrogoniometric method. The Journal of Bone and Joint Surgery.

[35] Kaddick C., Wimmer M.A. (2001). Hip simulator wear testing according to the newly introduced standard ISO 14242. Proceedings of the Institution of Mechanical Engineers: Part H, Journal of Engineering Tribology.

[36] Galvin A.L., Tipper J.L., Jennings L.M., Stone M.H., Jin Z., Ingham E. (2007). Wear and biological activity of highly crosslinked polyethylene in the hip under low serum protein concentrations. Proceedings of the Institution of Mechanical Engineers: Part H, Journal of Engineering in Medicine.

[37] Goldsmith A.A., Dowson D. (1999). A multi-station hip simulator study of the performance of 22 mm diameter zirconia-ultra-high molecular weight polyethylene total replacement hip joints. Proceedings of the Institution of Mechanical Engineers: Part H, Journal of Engineering in Medicine.

[38] Wang A. (2001). A unified theory of wear for ultra-high molecular weight polyethylene in multi-directional sliding. Wear.

[39] Turell M., Wang A., Bellare A. (2003). Quantification of the effect of cross-path motion on the wear rate of ultra-high molecular weight polyethylene. Wear.

[40] Lee K., Pienkowski D. (1998). Compressive creep characteristics of extruded ultra high molecular weight polyethylene. Journal of Biomedical Materials Research.

[41] Bigsby R.J.A., Hardaker C.S., Fisher J. (1997). Wear of ultra-high molecular weight polyethylene acetabular cups in a physiological hip joint simulator in the anatomical position using bovine serum as a lubricant. Proceedings of the Institution of Mechanical Engineers: Part H, Journal of Engineering in Medicine.

[42] Saikko V., Kostamo J. (2011). RandomPOD—a new method and device for advanced wear simulation of orthopaedic biomaterials. Journal of Biomechanics.

[43] Jin Z, Medley JB and Dowson D. Fluid film lubrication in artificial hip joints. Tribological research and design for engineering systems. In: Dowson D, Priest M, Dalmaz G, Lubrecht AA, editors, Proceedings of the 29th Leeds-Lyon symposium on tribology. Amsterdam: Elsevier; 2003. p. 237–256.

[44] Lee R.K., Korduba L.A., Wang A. (2011). An improved theoretical model of orientation softening and cross-shear wear of ultra high molecular weight polyethylene. Wear.

